# Multicenter randomized trial of deferred cytoreductive nephrectomy in synchronous metastatic renal cell carcinoma receiving checkpoint inhibitors: the NORDIC-SUN-Trial

**DOI:** 10.1186/s12885-024-11987-3

**Published:** 2024-02-24

**Authors:** Laura Iisager, Johanne Ahrenfeldt, Frede Donskov, Börje Ljungberg, Axel Bex, Lars Lund, Iben Lyskjær, Niels Fristrup

**Affiliations:** 1 Department of Molecular Medicine, Aarhus University Hospital, Aarhus, Denmark; 2 Department of Oncology, Southern Denmark University Hospital, Esbjerg, Denmark; 3 Department of Clinical Research, University of Southern Denmark, Odense, Denmark; 4 Department of Surgical and Perioperative Sciences, Umeå University, Umeå, Sweden; 5 Specialist Centre for Kidney Cancer, Royal Free Hospital London, London, England; 6 Department of Urology, Odense University Hospital, Odense, Denmark; 7 Department of Oncology, Aarhus University Hospital, Aarhus, Denmark

**Keywords:** Synchronous metastatic renal cell carcinoma, Immunotherapy, Deferred cytoreductive nephrectomy, Biomarkers, Translational research

## Abstract

**Background:**

Primary tumor removal by cytoreductive nephrectomy in synchronous metastatic renal cell carcinoma patients has been investigated in the context of various treatment regimens. Two randomized controlled trials investigated the role and timing of cytoreductive nephrectomy in the era of targeted therapy and demonstrated that upfront nephrectomy should no longer be performed when patients require systemic therapy. Superiority of checkpoint immunotherapy agents has led to a paradigm change from targeted therapies to immunotherapy-based first-line treatment in patients with primary metastatic disease; thus, deferred cytoreductive nephrectomy needs to be verified in the immunotherapy setting. Furthermore, a need exists for personalizing treatment choices for the individual patient to avoid unnecessary overtreatment.

**Methods/design:**

To explore the impact of cytoreductive nephrectomy in this patient group receiving checkpoint immunotherapy, we initiated a randomized, controlled trial comparing deferred cytoreductive nephrectomy with no surgery. The trial integrates a comprehensive translational research program with specimen sampling for biomarker analysis.

**Discussion:**

The trial aims to show that deferred cytoreductive nephrectomy improves overall survival in patients with synchronous metastatic renal cell carcinoma, and furthermore, to identify relevant biomarkers for personalized renal cancer management.

**Trial registration:**

ClinicalTrials.gov NCT03977571 June 6, 2019.

**Supplementary Information:**

The online version contains supplementary material available at 10.1186/s12885-024-11987-3.

## Background

### Cytoreductive nephrectomy in synchronous metastatic renal cell carcinoma patients

Around 20% of patients with Renal Cell Carcinoma (RCC) have synchronous metastatic disease at diagnosis, i.e. are diagnosed with a primary kidney tumor and metastases simultaneously. Patients presenting with metastatic disease at primary diagnosis have the worst prognosis due to underlying aggressive tumor biology and are often clinically challenging due to symptoms from either the primary tumor or the metastases [1, 2]. The critical question is whether the primary kidney tumor should be removed in patients with de novo, synchronous, mRCC.

In the clinic, mRCC patients are stratified into ‘favorable’, ‘intermediate’, and ‘poor’ prognostic risk groups according to 0, 1–2, or 3–6 international metastatic renal cell carcinoma database consortium (IMDC) risk factors, respectively, with the majority (95%) of de novo synchronous mRCC patients belonging to the intermediate or poor risk group [2, 3].

Despite the improvements that targeted treatment and checkpoint immunotherapies have brought to mRCC, response rates are still overall poor across patients (reviewed in [4]). Surgical resection of the primary tumor (cytoreductive nephrectomy, CN) upon metastatic spread at diagnosis is currently recommended following systemic therapy for patients with a good general health condition, few IMDC risk factors (maximum of three), and a response to systemic therapy [5, 6]. The concept is that CN combined with systemic therapy may result in no evidence of disease and eliminate a potential source of immunosuppressive or tumor-promoting growth factors [7–9]. On the other hand, if performed initially, there is a risk for operated patients never to receive or benefit from systemic therapy due to complications following CN or rapid tumor progression, due to having their commencement of systemic treatment delayed because of surgery complications, or due to hampering of the immune response following antibiotic treatment after surgery [10]. Recently, a post hoc analysis from the JAVALIN renal 101 phase 3 trial indicated that CN may improve OS in synchronous metastatic patients receiving tyrosine kinase inhibitor and immunotherapy (TKI-IO) treatment [11].

In the era of targeted TKI therapy two randomized trials, CARMENA [12] and SURTIME [13], have questioned the role and timing of CN in synchronous mRCC patients with results pointing towards an improved benefit upon a deferred approach [13]; except for poor risk patients [7]. The deferred CN approach ensures systemic therapy for all patients, avoids systemic treatment delay, and spares surgery in patients with progressive tumors [12]. Furthermore, baseline IMDC risk features are dynamic [14, 15] and the administration of systemic therapy may change deviant blood measures and performance status of the patient, and thus change the IMDC risk score, possibly increasing surgery eligibility. However, both studies investigating the CN and TKI combinatorial effect were underpowered and performed in a previous treatment era, and thus the effect of CN in synchronous mRCC needs to be further verified in the present checkpoint immunotherapy (IO) era. Retrospective analyses have reported benefits of CN in IO-treated patients [16], however as CN only is performed in patients with a good performance status the results may be biased. To test the effect of CN in IO-treated mRCC patients the randomized prospective trial; Deferred Cytoreductive Nephrectomy in Synchronous Metastatic Renal Cell Carcinoma: The NORDIC-SUN-Trial (NCT03977571), described here, has been initiated. We aim to test the effect of deferred CN in mRCC patients receiving combination IO (IO-IO) or TKI-IO, which is currently used as standard treatment for IMDC intermediate- and poor-risk mRCC patients. Concurrently, the PROBE trial (NCT04510597) is recruiting in the US, also trying to answer this clinical question, thereby underlining its importance in the management of primary metastatic RCC.

### Predictive biomarkers for precision medicine

There is a pressing need in renal cancer patient management for reliable and clinically accessible biomarkers to predict treatment response and surgery benefit to tailor the right treatment for the individual patient hereby minimizing negative consequences to patients while assuring optimal usage of health expenses.

Immunotherapy executes its antitumor probabilities by activating the patient's own immune system. However, only little is known about the immunomodulatory and biological effects of nivolumab combined with ipilimumab, as well as the new treatment combination of TKI-IO therapy in mRCC, with the IO-IO therapy having no effect in 20% of patients with their best objective response being progressive disease [17]. Consequently, we have an urgent need for biomarkers to assure we treat the right patients. Recent studies have indicated that the treatment response may be impacted by the composition of the immune T cells as well as the microbiota (reviewed in [18]). Moreover, circulating tumor DNA (ctDNA) presents a promising sensitive tool for prognostication, detection of relapse, and monitoring of treatment response as it captures intra- and inter-tumor heterogeneity, and correlates with tumor burden [19, 20]. RCC is genetically a multistep process (loss of 3p on the one allele, followed by mutations on the other allele in *VHL*, *PBRM1*, *BAP1*, *SETD2*, and subsequently loss of 9p and 14q), with increasing tumor aggressiveness by an increasing number of genetic events [21]. Thus, mapping the genetic events may predict which patients would benefit, or not benefit, from surgery and treatment. The present trial will integrate a comprehensive translational research program with various specimen sampling for biomarker analysis using fresh frozen tumor tissue, blood samples and rectal swabs, to personalize renal cancer treatment in the future.

## Methods/design

### Rationale

The deferred CN approach enables all patients to be treated with systemic therapy, restricting only patients benefitting from the therapy to receive surgery. However, current data are derived during the TKI era and only point towards a survival benefit for CN in the subgroup patients with a maximum of 3 IMDC risk factors. In this trial we aim to describe the effect of deferred CN in a contemporary patient population treated with IO-IO or TKI-IO therapy.

Furthermore, the genomic landscape of the tumor, tumor microenvironment composition, peripheral immune subsets and the microbiome may be of great importance to the efficacy of treatment and to select candidates for surgery. To facilitate this, we will collect patient specimens longitudinally during the trial.

### Objectives and endpoints

The aim of this study is to conduct an open, randomized, multicenter comparison trial, evaluating the effect of deferred CN compared with no surgery, following initial nivolumab combined with ipilimumab or a TKI-IO-combination, in synchronous mRCC patients with ≤ 3 IMDC risk features and absence of progressive disease at metastatic sites.

### Primary endpoint

The primary endpoint is overall survival (OS) compared between synchronous mRCC patients receiving a deferred CN and patients not receiving surgery.

### Secondary endpoints include

- Progression-free survival (PFS)
- Time to subsequent systemic therapy (TST)
- Objective response rate (ORR) and disease control rate (DCR) per RECIST 1.1
- Surgery complications and 30-day mortality (Clavien-Dindo)
- Fractional percentage of tumor volume to survival outcome
- Percentage of pathological response to survival outcome
- Frequency of patients meeting the randomization criteria
- OS, PFS, TST, and ORR in patients not meeting the randomization criteria and in patients with clear cell and non-clear cell histology

### Exploratory endpoints

- Immune cells, tumor cells, ctDNA, and the microbiome will be analyzed and compared with OS, PFS, TST, ORR, pathological response, DCR, and safety.<div class="NodiCopyInline">Immune cells, tumor cells, ctDNA, and the microbiome will be analyzed and compared with OS, PFS, TST, ORR, pathological response, DCR, and safety.</div>

### Study design

NORDIC-SUN is a multicenter open label randomized trial investigating the impact of CN in synchronous mRCC following 3 or 6 months of immunotherapy-based first-line treatment. Patients will be stratified by 1:1 allocation into Arm A (deferred CN) and Arm B (no surgery) based on criteria of surgical resectability, ≤ 3 IMDC risk features, and absence of progressive disease at metastatic sites. The remaining patients not eligible for randomization, referred to as Arm C, will be described, but are not a part of the main analysis.

**Fig. 1:**
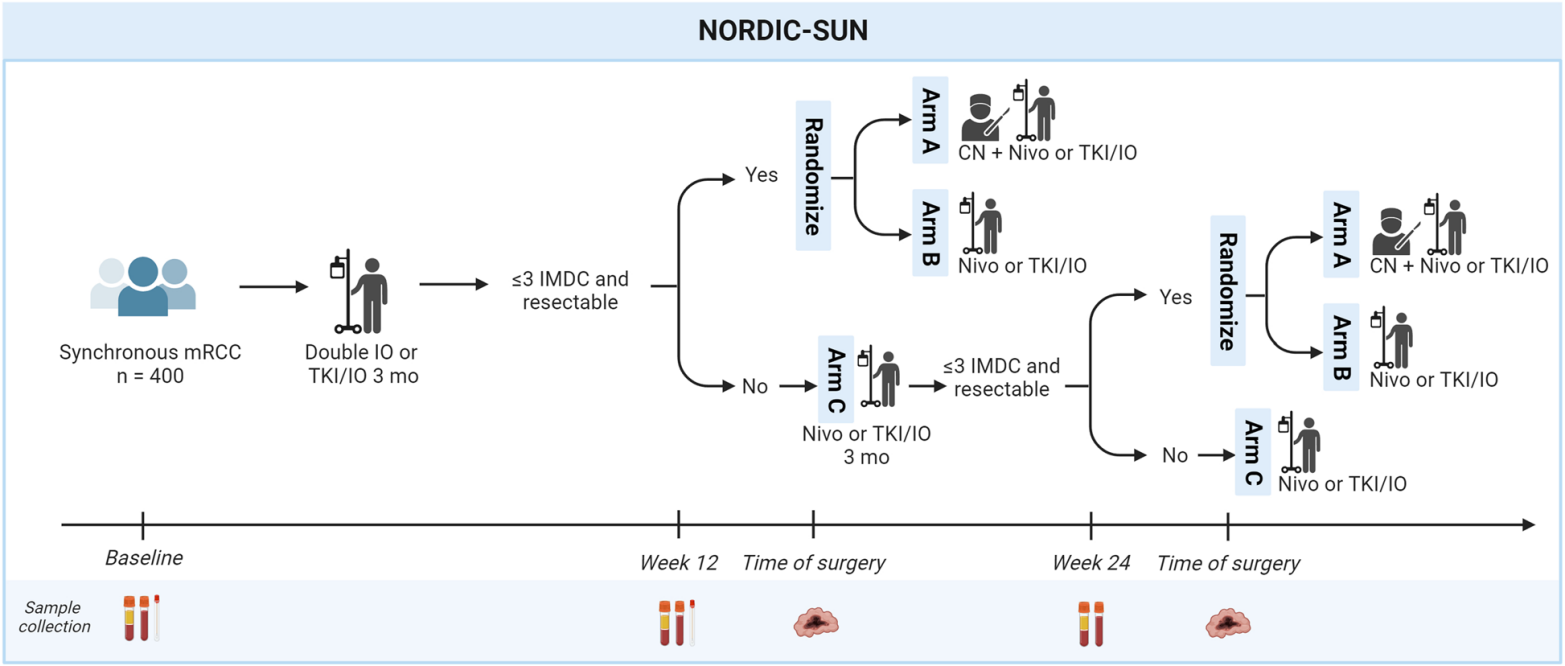
Study design. Arm **A**: Deferred CN followed by nivolumab or TKI-IO therapy. Arm **B**: No surgery, nivolumab or TKI-IO therapy. Arm **C**: Patients not eligible for randomization into Arm **A** or **B**. CN: Cytoreductive Nephrectomy; IO: Immunotherapy; mRCC: metastatic Renal Cell Carcinoma; Nivo: Nivolumab; TKI: Tyrosine Kinase Inhibitor. Generated with BioRender.com

The study design is outlined in Fig. 1.

### Study population

The trial will include 400 mRCC patients with synchronous disease. Participating centers will be University Hospitals in Denmark and the Nordic Countries, and possibly a few European sites. The coordinating center, which includes a data manager, is placed at Aarhus University Hospital, Denmark, managing the trial in close collaboration with the PI and the steering committee. Collaborating centers report to the coordinating center.

### Detailed enrollment criteria are listed below

- *Inclusion criteria:*
  - Synchronous mRCC proven with a core needle biopsy (all histological subtypes are acceptable) and measurable as per RECIST v 1.1.
  - Age > 18.
  - Patients referred to standard immunotherapy-based first-line treatment according to the recommendations by the European Medicines Agency and the national health authorities. Both IO-IO and TKI-IO are considered as a standard treatment.
  - Females not pregnant. Fertile patients must use effective means of contraception.
  - Life expectancy > 4 months.
  - Karnofsky Performance status > 70.
  - Laboratory values acceptable for IO-IO or TKI-IO treatment.
  - Able to speak and understand native language.
- *Exclusion criteria:  *
  - Prior systemic treatment for mRCC.
  - Other cancer diagnosis within three years (except in situ basal cell carcinoma and radically treated localized prostate cancer with undetectable prostate-specific antigen).
  - Major surgical procedure, open surgical biopsy, or significant traumatic injury within 14 days prior to enrollment, or non-healed lesion.
  - Clinically significant (i.e. active) cardiovascular disease for example cerebrovascular accidents (< 6 months before inclusion), myocardial infarction (< 6 months before inclusion), unstable angina, New York Heart Association grade II or greater congestive heart failure.
  - No symptomatic brain metastasis requiring systemic corticosteroids (> 10 mg daily prednisone equivalent).
  - Recent (within the 30 days prior to inclusion) treatment with another investigational drug.
  - Any active or recent history of a known or suspected autoimmune disease or recent history of a condition that requires systemic corticosteroids (> 10 mg daily prednisone equivalent) or other immunosuppressive medications, excluding inhaled steroids and local cutaneous steroids. Subjects with vitiligo or type I diabetes mellitus or residual hypothyroidism due to autoimmune thyroiditis only requiring hormone replacement, psoriasis not requiring systemic treatment are permitted to enroll.
  - Known hypersensitivity to monoclonal antibodies.
  - Known history of testing positive for human immunodeficiency virus or known acquired immunodeficiency syndrome (AIDS).
  - Any positive test for hepatitis B- or C-Virus, indicating acute or chronic infection.

### Randomization and masking

Patients are randomized 1:1 into the two treatment arms:

- ARM A: 3 months of IO-IO or TKI-IO followed by deferred CN, followed by maintenance nivolumab or a TKI-IO-combination.
  ARM B: No surgery, 3 months of IO-IO or TKI-IO followed by maintenance nivolumab or a TKI-IO-combination.

Patient eligible for surgery is randomized by a local project nurse using a computer-generated allocation sequence and is not blinded due to the circumstances; surgery versus no surgery. In Arm A patients are treated according to standard protocol with first-line IO-IO or TKI-IO combination treatment followed by a CN. In Arm B patients are receiving standard IO-IO or a TKI-IO combination first-line treatment (Fig. 1). Patients will be stratified by treatment choice (IO-IO vs TKI-IO).

The remaining patients, referred to as Arm C, not eligible for randomization due to > 3 IMDC risk factors, progressive disease at metastatic sites, poor performance or a non resectable tumor will undergo three months of nivolumab or a TKI-IO combination and then be evaluated again after another 3 months. If still not eligible for randomization, the patients will continue nivolumab maintenance and TKI-IO combination and be followed on survival.

### Study procedure

Patients included in the NORDIC-SUN trial are stratified according to institution, treatment choice, number of IMDC risk factors, and combined elevated neutrophil–lymphocyte ratio and hyponatremia.

All patients will receive systemic IO-IO or TKI-IO immediately after inclusion. After three months of nivolumab combined with ipilimumab or a TKI-IO combination, the patient will be discussed for resectability at the multidisciplinary meeting (MDT), whether the patient is eligible for CN. Patients with ≤ 3 IMDC risk factors and deemed suitable for CN including an acceptable performance status and absence of progression at metastatic sites will then undergo randomization. Patients deemed not suitable for surgery or have > 3 IMDC risk features at the 3 month evaluation continue systemic therapy for 3 months, followed by a 2nd evaluation 6 months after inclusion. Patients with ≤ 3 IMDC risk factors and deemed suitable for CN at 2nd evaluation will then undergo randomization. Patients deemed not suitable for surgery or have > 3 IMDC risk features at the 6 month evaluation continue systemic therapy (Arm C). Nivolumab may continue until unacceptable toxicity or total treatment length of two years from inclusion. Allocating patients to Arm C promotes participant retention, securing outcome data of patients not randomized.

### Status

Inclusion began right before the Corona epidemic set in. The study was therefore paused and has now been re-initiated in January 2023. Presently, 40 patients are included (10% accrual) and the study will continue until full accrual (400 patients) have been reached in 2026, with a follow-up period of additional three years. The primary endpoint is expected to be reached and ready for publication in 2029.

### Sample collection

All patients will have blood samples collected at baseline, week 12, and week 24. Rectal swabs are taken as a proxy for stool samples at baseline and week 12. At baseline, a fresh frozen tumor core biopsy is seeked to be obtained and for Arm A biopsies from the nephrectomy specimen will be fixed and stored according to the Danish Cancer Biobank for future analyses. All samples will be anonymously labeled with a pseudo patient study identifier. See Fig. 1.

### Experimental plans

- ctDNA will be detected and measured in plasma samples using sequence-based methods.
- Bacterial species will be identified using 16 s ribosomal RNA gene sequencing and linked to response data to determine the role of the colonic microflora in immunotherapy response.
- The beta chain gene repertoire of T cell receptors will be sequenced to determine T cell clone dynamics and composition.
- Whole exome and genome sequencing will be applied to explore and validate predictive markers of treatment response.
- RNA-sequencing will be performed to investigate the association between molecular subtypes, immune subtypes, and gene expression signatures of cellular processes and treatment response.

### Statistical analyses

#### Sample size determination

Sample size calculations are based on simulated survival data and cox regression. With the assumption that 60% of patients meet the randomization criteria, and to demonstrate a 50% improvement in OS, with a 3-year follow-up, a sample size of 400 patients is needed to achieve 80% power at a 5% significance level. Hence, 400 patients will be included in NORDIC-SUN with the estimation of 240 patients (60%) being randomized (120 in each arm) and 168 occurred deaths upon reach of appropriate power.

#### Data analysis

Difference in primary and secondary endpoints between the two treatment arms will be tested with a two-sided log rank test at the 5% alpha level. Statistical methods including Kaplan–Meier and cox proportional hazard regression will be performed adjusted and unadjusted for the stratification factor. Furthermore, linear regression, logistic regression, and machine learning approaches (e.g. Random Forest, Neural Networks, and Gradient Boost models) will be applied in an exploratory manner to identify any correlation between molecular measures and clinical outcome parameters.

### Biobank

Blood, tissue, and fecal swab samples collected during the trial will be stored in the Bio- and Genome Bank, Denmark. Residual blood and tissue biopsies as well as clinical and sequencing data will be transferred to the Renal Cancer Research Biobank at Aarhus University Hospital for future research, where it will be saved and stored in a pseudonymized form.

### Dissemination

Any protocol modifications will be reported timely to the relevant authorities, investigators/centers, and if necessary, patients. No protocol modification is effected before the necessary approval have been given. Results from the NORDIC-SUN trial will be published in peer-reviewed scientific journals.

## Discussion

For synchronous mRCC, the question whether the primary tumor responds just as well as the remaining systemic disease to combinational IO-IO or TKI-IO treatment remains. Moreover, the timing of surgery versus systemic treatment is still debated. The impact of debulking the primary tumor in patients with synchronous mRCC has been evaluated in two prospective randomized studies in the TKI era. The SURTIME trial questioned the timing of CN, upfront or deferred, and reported that unnecessary surgery could be avoided through pre-treatment with sunitinib to identify patients inherently resistant to systemic targeted therapy [13]. The CARMENA trial tested the benefit of initial nephrectomy and showed no benefit of upfront nephrectomy over sunitinib alone [12]. As 20% of the patients in the sunitinib alone arm in CARMENA had a deferred CN, both SURTIME and CARMENA can be regarded as proof-of-concept trials demonstrating that patients who require systemic therapy should receive drug therapy first with the option to be offered deferred CN after disease consolidation. Now that the preferred treatment for mRCC has changed to favoring doublet IO or TKI-IO, the role of deferred CN in this setting is yet to be determined.

In the presented randomized clinical trial, the NORDIC-SUN trial, the role of surgery will be determined in both poor and intermediate risk patients. It is expected that the initial first-line of IO-IO or TKI-IO treatment will reduce the IMDC risk score for some patients, thereby making more patients eligible for surgery. Similarly to the NORDIC-SUN trial, the Prospective Randomized Open Blinded Endpoint (PROBE) trial (NCT04510597) has been initiated to evaluate if CN provides an overall survival benefit in patients responding to first-line systemic therapy (objective response or stable disease) [22]. The PROBE trial tests CN and systemic therapy (IO-IO or TKI-IO) against systemic therapy alone. Enrollment started in 2021 and the study is expected to be completed in 2033. Comparison of the results from these two trials would be warranted.

We hypothesize that deferred CN after initial immunotherapy-based first-line treatment will improve OS in patients with synchronous metastatic RCC and ≤ 3 IMDC risk features. Furthermore, this study will identify potential predictive and prognostic biomarkers based on immune subsets in blood and tumor, gene expression, tumor mutational burden, overall genetic alterations, ctDNA, and gut microbiome. Our findings aim at helping clinicians to stratify patients more accurately for surgery and/or immunotherapy-based combinational treatment.

## Conclusion

Patients with de novo, synchronous metastatic disease are clinically challenging. The NORDIC-SUN trial is a randomized clinical trial evaluating the effect of deferred CN during first-line immunotherapy doublet or TKI-IO treatment only in synchronous metastatic RCC patients. The trial will identify potential predictive and prognostic biomarkers, thereby seeking to personalize the treatment for each patient.

### Supplementary Information

**Additional file 1.** Checklist for SPIRIT guidelines.

## Data Availability

Future data from the trial will be available after publication of trial results from the corresponding author on reasonable request.

## Abbreviations

CN: Cytoreductive nephrectomy
ctDNA: Circulating tumor DNA
DCR: Disease control rate
IMDC: International metastatic renal cell carcinoma database consortium
IO: Immunotherapy
PFS: Progression free survival
RCC: Renal cell carcinoma
mRCC: Metastatic renal cell carcinoma
Nivo: Nivolumab
ORR: Objective response rate
OS: Overall survival
TKI: Tyrosine kinase inhibitor
TST: Time to subsequent systemic therapy
